# The Role of the MiR-181 Family in Hepatocellular Carcinoma

**DOI:** 10.3390/cells13151289

**Published:** 2024-07-31

**Authors:** Jinbiao Chen, Ken Liu, Mathew A. Vadas, Jennifer R. Gamble, Geoffrey W. McCaughan

**Affiliations:** 1 Liver Injury and Cancer Program, Cancer Innovations Centre, Centenary Institute, Sydney Medical School, Faculty of Medicine and Health, The University of Sydney, Camperdown, NSW 2050, Australia; ken.liu@health.nsw.gov.au; 2 Royal Prince Alfred Hospital, Missenden Road, Camperdown, NSW 2050, Australia; 3 Vascular Biology Program, Healthy Ageing Centre, Centenary Institute, Sydney Medical School, Faculty of Medicine and Health, The University of Sydney, Camperdown, NSW 2050, Australia; m.vadas@centenary.org.au (M.A.V.); j.gamble@centenary.org.au (J.R.G.)

**Keywords:** non-coding RNA, microRNA targets, Cbx7, anti-miR, transcriptional regulation, Akt pathway, MAPK pathway, microRNA knockout, TGF-beta

## Abstract

Hepatocellular carcinoma (HCC) is the fourth-leading cause of cancer-related death worldwide. Due to the high mortality rate in HCC patients, discovering and developing novel systemic treatment options for HCC is a vital unmet medical need. Among the numerous molecular alterations in HCCs, microRNAs (miRNAs) have been increasingly recognised to play critical roles in hepatocarcinogenesis. We and others have recently revealed that members of the microRNA-181 (miR-181) family were up-regulated in some, though not all, human cirrhotic and HCC tissues—this up-regulation induced epithelial–mesenchymal transition (EMT) in hepatocytes and tumour cells, promoting HCC progression. MiR-181s play crucial roles in governing the fate and function of various cells, such as endothelial cells, immune cells, and tumour cells. Previous reviews have extensively covered these aspects in detail. This review aims to give some insights into miR-181s, their targets and roles in modulating signal transduction pathways, factors regulating miR-181 expression and function, and their roles in HCC.

## 1. Introduction

Hepatocellular carcinoma (HCC) is the fourth-leading cause of cancer-related death worldwide [1,2,3]. More than 50% of patients are diagnosed with advanced HCC. Unfortunately, only a few first-line drugs are available for patients with advanced HCC, such as sorafenib, lenvatinib, and the combination of atezolizumab and bevacizumab. Due to the high mortality rate and unmet treatment options in HCC patients, discovering and developing novel systemic treatment options for HCC is a vital unmet medical need [4,5,6,7,8,9].

Among the numerous molecular alterations in HCCs, microRNAs (miRNAs) have been increasingly recognised for their crucial roles in hepatocarcinogenesis. After being extensively studied over the past two decades, the precise significance and function of the miRNAs in HCC formation and progression remain elusive [10,11,12,13]. We and others have recently revealed that members of the microRNA-181 (miR-181) family were up-regulated in some, if not all, human cirrhotic and HCC tissues. Up-regulated miR-181 induced hepatocyte epithelial–mesenchymal transition (EMT), promoting HCC progression [10,14,15,16,17,18,19]. MiR-181 has been widely reported to play vital roles in governing the fate and function of endothelial cells, fibroblasts, and immune cells [10,12,20,21,22,23,24], which are all involved in cancer initiation and progression to a certain extent. Previous reviews have extensively covered these aspects in detail. Herein is a concise review of miR-181, its targets and regulations, and especially its overlooked significance in HCC.

## 2. MiRNAs and the miR-181 Family

MiRNAs are short non-coding RNA (ncRNA) transcripts that are not translated into proteins [25,26,27,28,29,30]. They are typically 20–24 nucleotides in length and play crucial roles in gene expression regulation by targeting mRNA transcripts. The microRNA database, i.e., miRBase (http://www.mirbase.org), currently records 1917 human miRNA precursors, encompassing over 2600 mature miRNAs. Bioinformatics analysis reveals more than 60% of human protein-coding genes contain conserved miRNA-binding sites, indicating their potential regulatory interactions [31,32,33,34]. Remarkably, a single miRNA can regulate multiple mRNA targets, and reciprocally, multiple miRNAs can target a single mRNA, significantly expanding their functional impact [32,33].

**Figure 1:**
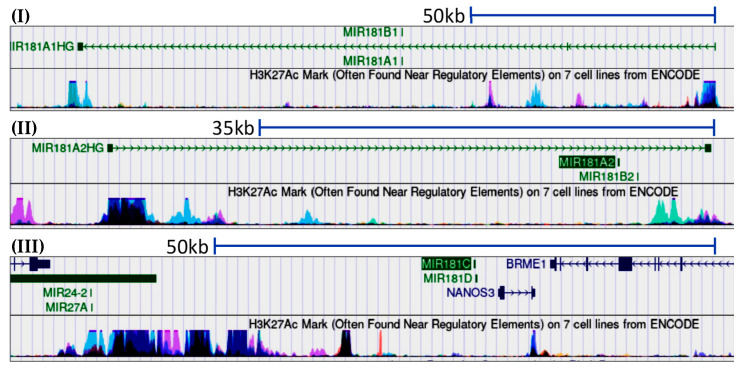
Chromosomal position of the three miR-181 clusters. (**I**) Mir181A/B1 and (**II**) mir181A/B2 transcription start sites (TSS) have been mapped to 78.3 kb and 34.0 kb upstream of the mature miRNAs, respectively, consistent with the position of H3K27Ac (in blue/pink), which is associated with the higher activation of transcription [38]. (**III**) The putative TSS of miR181C/D might be 9 kb upstream of the miR-181C/D precursor. Please note that the clusters are not drawn to scale.

The miR-181 family consists of six highly conserved members: miR-181A1 (Figure 1I), miR-181A2 (Figure 1II), miR-181B1 (Figure 1I), miR-181B2 (Figure 1II), miR-181C (Figure 1III), and miR-181D (Figure 1III). All mature miR-181s share the same “seed” sequence, “ACAUUCA” [20], but their mature sequences are different. MiR-181A1 and miR-181A2 are identical in their mature sequences, but they are located on different chromosomes, and the situation is similar for miR-181B1 and miR-181B2 (Figure 1I,II). Indeed, they are independently derived from three clusters located on three chromosomes. MiR-181A/B1 is found in an intron of a non-coding RNA host gene (MIR181A1HG) on chromosome 1 in mice and humans [13,35,36]. MiR-181A/B2 is found in an intron of the NR6A1 gene on chromosome 2 in mice and chromosome 9 in humans [37]. MiR-181C and miR-181D are found in an uncharacterized sequence on chromosome 8 in mice and chromosome 19 in humans and are transcribed independently [20].

The targets of each miR-181 member are largely overlapping, as all mature miR-181s share the same “seed” sequence [10,20,33,39,40]. On the other hand, the 3p and 5p strands of each miR-181 member are different, albeit by only a few nucleotides [10,20,39]. Different pre-miRNA loop nucleotides also regulate the distinct activities of miR-181 members [39]. Thus, each miR-181 member also targets many genes, leading to multiple potential functions [39,40].

Mice lacking each of the three miR-181 clusters have been successfully generated [41,42,43,44,45]. Notably, the greater the deletion of the three miR-181 clusters, the more significant the reduction in mouse size, weight, and vitality [10,20,41,43]. The generation of triple-knockout mice that lack all three miR-181 clusters has proven unattainable due to embryonic lethality. These findings from mouse genetic studies underscore the crucial role of miR-181 family members in regulating cell growth and development [10,20,41,42,43].

## 3. Targets of miR-181

### 3.1. Database of miRNA Targets

MiRNAs primarily regulate gene expression by targeting gene transcripts. Understanding the potential function of a specific miRNA often begins with exploring its targets. Several miRNA target prediction tools have been developed, but due to the complex mechanism underlying miRNA action, the predicted targets often vary [46,47]. Some examples of widely used miRNA target prediction tools are (i) TargetScan (v8.0): TargetScan is a popular miRNA target prediction tool that incorporates information about miRNA seed regions and evolutionary conservation to predict potential target sites [40,48]; (ii) miRanda (v3.3a): miRanda utilizes a scoring algorithm that considers sequence complementarity and conservation to predict miRNA targets [49]; (iii) PicTar: PicTar predicts miRNA targets by integrating sequence complementarity, conservation, and the presence of miRNA target site clusters [50]; (iv) RNAhybrid (v2.2.1): RNAhybrid employs a thermodynamic model to predict the energetically most favourable interaction between an miRNA and its target site [51]; (v) PITA: PITA (miRNA target prediction by base pairing probability) predicts miRNA targets by calculating the base pairing probability between miRNAs and potential target sites [52]; (vi) DIANA-microT (v2023): DIANA-microT employs a machine learning approach to predict miRNA targets by integrating multiple features, such as site accessibility and seed region conservation [53]; and (vii) miRDB (v6.0): miRDB is an online database that provides miRNA target predictions based on a machine learning algorithm trained on experimental data [54]. While TargetScan, miRanda, and DIANA-microT are popular and perform well, their false positive and false negative rates are still very high.

Thus, experimental evidence is required to validate miRNA–target interactions [55]. MiRNA research has yielded a growing number of experimentally validated miRNA–target interactions since 1993. These valuable findings have been compiled and summarized in several databases, including miRWalk (v3) and miRTarBase (v9.0 beta) [56,57]. The miRTarBase is a fully manually curated online database that stores validated miRNA targets. The more validated experimental data that accumulate, the more miRNA targets will be verified.

It should also be noted that phenotypic changes of disrupting single miRNA–target interaction are often subtle for the following reasons: (i) most (>90%) of gene transcripts are targeted by more than one miRNA, so most miRNA targets would be down-regulated by less than 50%; (ii) the protein expression of most miRNA targeted genes can vary by two-fold without causing detectable consequences; and (iii) miRNA targets are regulated by many regulatory elements, i.e., buffering effects of the regulatory network [32].

### 3.2. MiR-181 Targets and Their Roles in Modulating Signal Transduction Pathways

According to TargetScan analysis, it has been predicted that 1371 human transcripts harbour conserved miR-181-5p binding sites. These transcripts collectively contain 1694 conserved binding sites and 960 poorly conserved binding sites. On average, each transcript possesses approximately two binding sites. In the miRTarBase database, there are 2556 documented instances of miR-181, including 1350 validated miR-181 (both miR-181-5p and miR-181-3p) targets. After comparing the predicted miR-181 targets with the validated miR-181 targets, only 435 of the targets overlapped. This discrepancy suggests the limitations of miRNA target predictors due to the intricate mechanisms governing the actions of miRNAs. Interestingly, a new study found an alternative seed match and identified a distinct set of miR-181 targets within the coding sequence of genes, revealing that miR-181 acts primarily through RNA destabilization and translational inhibition [58], but more studies are needed to determine whether the results of this study can eliminate the discrepancy mentioned above.

The miR-181 family has been extensively studied. There are more than 2600 original papers in PubMed that discuss miR-181 to some extent. MiRNA overexpression, knockdown, or BlockmiR are often used to investigate the function of a specific miRNA [20,59]. It is complex to dissect the miR-181 family because it consists of six members, and each member exhibits different expression levels in different types of cells [39,41,42,44,60,61,62]. Furthermore, each member of the miR-181 family shows variable expression levels within a single cell [60]. Thus, miR-181 overexpression experiments should be carefully designed and interpreted, as any of the miR-181 family members could also affect targets of other miR-181 family members [20]. The deletion of miR-181s is more likely to accurately reveal the functions of an miR-181 member. Results obtained with selective inhibition of one miR-181 member by anti-miRs or similar techniques are also difficult to interpret because the effects of these anti-miRs could not be ruled out from silencing other miR-181 family members [20]. Nonetheless, hundreds of genes have been reported as targets of miR-181 [13], for example, Smad7 [63,64,65,66], TGFBR3 [67], TGFBR1 [68,69], Sema3A [70], Bim, ATM, Cbx7 [60,71,72,73], TIMP3 [17,19,74,75], CDH1, CDNK1B, and BCL2 [13]. Interestingly, many of these are important components of signalling pathways, such as TGF-β, PI3K/AKT, MAPK/ERK, Notch, and NFκB, as summarized in several reviews [10,11,13,20]. The role of miR-181 in regulating TGF-β, PI3K/AKT, and MAPK/ERK signalling is briefly discussed below.

A biological process known as epithelial–mesenchymal transition (EMT) involves the loss of polarity and cell–cell adhesion in epithelial cells, leading to the acquisition of migratory and invasive characteristics, ultimately transforming epithelial cells into mesenchymal stem cells [14,76,77,78,79,80]. The EMT plays a significant role in tissue fibrosis, cell migration (tumour metastasis), and the development of chemoresistance in cancer [14,77,79,81,82]. TGF-β has been well documented to induce EMT in many cells, including hepatocytes and tumour cells [14,77,79,82]. Interestingly, miR-181 targets many components of the TGF-beta signalling pathway, e.g., Smad7 [63,64,65,66], TGFBR1 [64,65,68,69,83], and TGFBR3 [67,84]. It has been widely reported that either TGF-β or miR-181 can induce EMT, but the detailed underlying molecular mechanisms responsible for the interaction between TGF-β and miR-181 and their roles in EMT are yet to be fully elucidated. TGF-β suppresses the early stages of tumour development by inducing cell-cycle arrest and apoptosis, but it later promotes tumour progression by increasing proliferative and anti-apoptotic signals via transactivating PI3K/AKT, MAPK/ERK, or other mitogenic pathways [60,85,86]. Our studies and others indicate that miR-181 may similarly play a dual role in the control of proliferation and apoptosis [13,60].

The PI3K/AKT signalling is a ubiquitously growth factor–regulated network. Its aberrant activation often impairs the proper control of cell growth, survival, and metabolism [87]. This pathway has been well documented to be regulated by miR-181 [42,62,72,88,89,90,91,92,93,94,95,96,97,98,99]. MiR-181 targets many components in the PI3K/AKT signalling pathway, such as PTEN, in vitro and in vivo [42,62,72,88,100]. The miR-181-PI3K/AKT axis conceivably plays a vital role in development, regeneration, immune homeostasis, and cardiac remodelling [42,62,100,101]. However, the precise role of miR-181 in regulating the PI3K/AKT signalling network in cancer cells has not been conclusively established, particularly considering the significant influence of various potent exogenous growth stimuli.

The highly conserved MAPK/ERK signalling pathway plays crucial roles in various biological events, including cell proliferation, differentiation, and death [102,103,104]. Gene mutations in the MAPK/ERK signalling network are common in many human tumours [102,103,104]. Approximately one-third of all human cancers, including pancreatic, lung, colorectal, and ovarian cancers, are driven by mutations in the RAS oncogenes (KRAS, HRAS, and NRAS), which are upstream molecules in the MAPK/ERK signalling pathway [102,103,104]. MiR-181 could be a tumour suppressor by reducing Ras signalling [102]. However, more reports show that miR-181 enhances the MAPK/ERK signalling, for example, by targeting DUSP6 [105,106,107,108,109]. Deletion of miR-181a/b1 suppressed the initiation and progression of KRAS mutant–induced primary lung and pancreatic cancer in mice, convincingly demonstrating that miR-181a/b1 mediates Ras signalling. It is unclear whether miR-181s and members of the Ras pathway form a feedback loop.

## 4. Factors Regulating miR-181 Expression and Function

The vital steps in miRNA biogenesis, such as the processing of miRNA precursors by Dicer, Drosha, and RISC loading, have been largely elucidated [110,111,112,113]. The expression levels and functions of individual miRNAs are regulated by chromosomal alterations (e.g., genetic changes in cancers), transcriptional regulation, epigenetic regulation, post-transcriptional regulation, and competing endogenous RNAs (e.g., miRNA sponging) [110,111,112,113,114,115,116]. The expression of miR-181s is regulated by BMP/TGF-β [13,14,19,63,64,65,67,69,70,83,117], WNT/β-catenin [13,16,118], VEGF signalling, and JAK/STAT3 [88], as well as being sponged by lncRNA SNHG7 and ITGA1 mRNA [73,96,119]. They are explained further below.

Many studies have consistently demonstrated that TGF-β increased the expression levels of miR-181s in multiple cells, including liver cancer cells [13,14,19,63,64,65,67,69,70,83,117]. However, only a few studies examined the transcriptional regulation of miR-181s [120,121]. Lutz CT and his team have successfully mapped human MIR181A1B1 and MIR181A2B2 transcription start sites (TSS) to 78.3 kb and 34.0 kb upstream of the mature miRNAs, respectively. They also identified transcription factor–binding sites of miR-181a/b1 and miR-181a/b2 in 51–201 nucleotides (nt) and 150–301 nt upstream of their TSS, respectively [120]. It has been shown that SMAD3 and SMAD4 could transactivate miR-181a/b2 promoter [120]. GATA3 has also been proposed to transactivate miR-181a/b1 promoter in mutant KRAS–induced cancers, in which TGF-β signalling was involved [121].

It has been reported that the expression level of miR-181 is up-regulated by Wnt/β-catenin signalling, which plays a vital role in the pathogenesis of multiple cancers, including HCC [13,16,118]. It has been reported that the promoter of miR-181a/b2 contains several TCF/LEF binding sites [118]. Interestingly, miR-181s target NLK, TIMP3, and GSK3β, all of which are negative regulators of Wnt/β-catenin signalling [16,74,122], indicating miR-181s and the Wnt/β-catenin signalling may form a positive feedback loop.

In addition to miRNAs, other ncRNAs, such as long non-coding RNAs (lncRNAs) and circular RNAs (circRNAs), have recently emerged as important regulators of miRNA function [25,26,27,28,29,30]. The function of miR-181s could be blocked by lncRNAs and circRNAs such as ANRIL, AFAP1-AS, ZEB1-AS1, ITGA1, Lnc-Nr6a1, CRNDE, circ-ANAPC7, cSMARCA5, and circHMGB2, which act as competing endogenous RNAs (ceRNAs) or miRNA sponges [37,73,75,119,123,124,125,126,127,128,129,130,131,132,133,134]. For example, lncRNA-ANRIL has been proposed to block the inhibitory effects of miR-181s on HMGB1, Sirt1, Prox1, and NFκB, especially in cardiovascular diseases [123,128,130,131,132,133,134].

## 5. Roles of miR-181 Family in Hepatocellular Carcinoma

### 5.1. Insights into the Role of miR-181 Family in HCC: Current Discoveries

MiRNAs are extensively recognised for their significant roles in liver diseases, as evidenced by numerous studies [11,14,15,16,17,18,19,60,78,92,96,101,135,136,137,138,139,140,141,142,143,144,145,146,147,148,149,150,151]. Their dysregulated expression has been identified in hepatocellular carcinoma (HCC) and is directly associated with tumour initiation and progression [136,147,148,149,150,151,152].

Since Ji J. et al. first reported the role of miR-181a in liver cancer stem cells in 2009 [16], many studies have further confirmed the impact of the miR-181 family in liver disease and liver cancer, albeit via various miR-181 target genes (Table 1) [14,15,16,17,18,19,60,75,96,118,137,153,154,155,156,157,158,159,160,161,162,163,164,165,166]. The importance of miR-181s in liver cancer has been much obscured by a large body of literature reporting the role of other miRs in hepatocarcinogenesis [136,147,148,149,150,151,152]. As such, the role of miR-181s in HCC has been largely unrecognized [18,147,148,152,167,168,169,170]. This can be attributed to many reasons, such as the inconsistent expression profiles of miR-181s in HCC [13,60,136,153], the heterogeneity of HCC [2,3,9], the dual regulatory effects of miR-181 on cancer formation [13], the different role of miR-181s in multiple cells [10,12,20,21,22,23,24], and the complexity of the miR-181 family, as described in Section 3.2 [20].

In 2009, it was reported for the first time that the expression of the miR-181 family was notably elevated in HCC, typically in liver cancer stem cells [16]. Its elevation played a pivotal role in sustaining the population of liver cancer stem cells by inhibiting CDX2, GATA6, and NLK [16]. Interestingly, TGF-β was shown to induce the upregulation of miR-181b, which in turn promoted HCC formation by down-regulating TIMP3 [19]. We found that miR-181a is one of the most amplified miRNAs during TGF-β-induced hepatocyte epithelial–mesenchymal transition (EMT) and is upregulated in fibrotic liver tissues and HCC samples from humans and mice [14].

The importance of miR-181a in human HCC was further enhanced by a seminal study using TCGA-LIHC human samples, where miR-181a was expressed at much higher levels than any other member of the miR-181 family. This paper grouped HCC cases based on multiple molecular parameters, including genomic mutations, DNA methylation, DNA copy number, and mRNA and miR expression. HCC cases were divided into three clusters, i.e., iClusters 1, 2, and 3. Patients in iCluster 1 had the worst prognosis. Strikingly, miR-181a was highly expressed in iCluster 1 compared with iClusters 2 and 3 [60,171].

Global or conditional miR-181 knockout (KO) mice have been generated and made available to the public. They have been widely used to study the roles of miR-181 in immune cells [20,39,41,44,60,62,93,100,101,108,121,146,172,173,174,175]. These valuable genetically engineered mouse models (GEMMs) are just beginning to be used to study miR-181s in cancer [60,121]. Our subsequent experiments using these GEMMS firmly corroborated the vital role of miR-181a/b1 in promoting chemically induced hepatocarcinogenesis [60]. In our experiments, liver tumour size was significantly reduced by 90% in global KO (GKO) or liver-specific KO (LKO) mice of miR-181a/b1 but not in hematopoietic and endothelial lineage-specific KO mice. However, we also showed that tumour induction requires both hepatic and non-hepatic miR-181 overexpression [60]. We showed that EMT was partially reversed in GKO tumours, linking to earlier studies that showed that miR-181a induced hepatocyte EMT [14]. The results obtained from these GEMMs indicate that miR-181a/b1 in tumour cells, but not in tumour-associated cells, plays the dominant role in the formation of DEN-induced liver cancer [60].

We identified CBX7, a known target of miR-181a/b1, as upregulated in miR-181a/b1 KO tumours. Furthermore, key CBX7-regulated genes were also upregulated. Stable upregulated CBX7 expression in vivo (using AAV) inhibited liver tumour progression, whilst hepatic CBX7 deletion restored the progression of LKO liver tumours. Thus, miR-181a/b1 upregulation via CBX7 inhibition in the tumour cells—rather than non-tumour cells within the tumour microenvironment (TME)—led to liver tumour progression [60].

CBX7 protein is a component of the Polycomb repressive complex 1 (PRC1) [60,73,176,177]. CBX7 plays a dual role in oncogenesis [176,177,178,179,180,181,182,183]. It may contribute to cancer progression by inhibiting tumour suppressor genes such as INK4a/ARF [176,177,179,180,181,182,183,184]. However, it has been shown more often to suppress cancer progression by transcriptionally or epigenetically regulating the expression of related proteins, therefore regulating tumour cell proliferation, migration, and invasion [60,71,178,179,185,186,187]. In addition, CBX7 protein may interact with different non-coding RNAs (microRNAs, long non-coding RNAs, circular RNAs) [72,73,187,188]. Post-translational modifications and distinct functions of CBX7 isoforms may add additional functional layers of CBX7 complexity [189,190]. Importantly, the miR-181/CBX7 axis has been widely verified in cancer and stem cells [60,71,72,73,178,191].

According to the results of our animal experiments and analysis of human HCC datasets, CBX7 was clearly shown to inhibit (rather than enhance) liver cancer progression via targeting multiple genes, such as WNT10a, DUSP4, FGFR2, and CCNE1 [60].

In the analysis of these data, we also showed that low miR-181 combined with high CBX7 expression in HCC was associated with the best prognosis and vice versa, with high miR-181 and low CBX7 HCC having the worst prognosis [60]. Furthermore, gene expression profiles of miR-181a/b1 KO HCCs in mice overlapped with low-proliferative periportal-type human HCCs with a good prognosis [60,192]. Thus, miRT-181a/b1-deficient liver tumours may be an ideal model for low-proliferative periportal-type human HCC.

### 5.2. The miR-181 Family in HCC: Summary and Future Perspectives

In summary, the biological functions of miRNAs and their diagnostic, therapeutic, and prognostic value in liver cancer have been extensively studied to a certain extent [16,18,59,60,135,136,147,149,171]. MiR-181a/b1, especially miR-181a, plays a crucial role in the formation of liver cancer by directly controlling the fate and function of tumour cells [12,20,21,60,71,72,73]. MiR-181s play a crucial pathogenic role in the formation and maintenance of stem cells, including liver cancer stem cells, deserving more studies to explore this aspect [16,17,45,72,99,144,157]. It will be very helpful to further thoroughly examine the expression levels of miR-181 members in different subtypes of HCC and different components of TME in humans. To do that, novel technologies are desired to effectively dissect the heterogeneity of HCC, the complexity of the miR-181 family, and their interactions. The emerging technologies, such as single-cell and spatial multi-omics technologies, including miRNA or miRNA-mRNA co-sequencing, may bring more clarity to miR-181s in human HCC [193,194,195].

**Figure 2:**
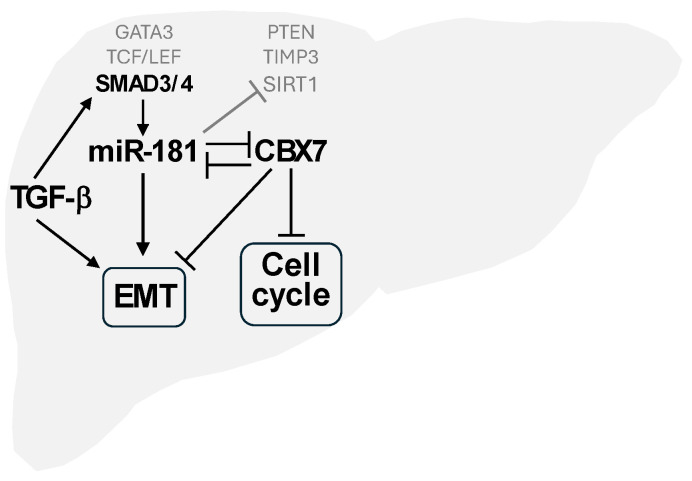
The TGF-β-miR-181-CBX7 axis. Arrow end lines mean activating, inducing, promoting; blunt end lines mean inhibiting, blocking, or reducing. EMT: epithelial–mesenchymal transition. Pathways such as Wnt-TCF/LEF-miR-181-CDX2/GATA6/NLK and KRAS-GATA3-miR-181a/b1 are partially shown. Several miR-181 targets other than CBX7 are also shown (in grey).

Among the signalling pathways modified by the miR-181 family [10,11,13,20], TGF-β-induced EMT signalling has been extensively explored and confirmed to be regulated by the miR-181 family. However, the detailed molecular mechanisms responsible for miR-181-induced EMT are largely unknown. CBX7 also plays a vital role in the regulation of EMT [60,76,185,186,196,197,198]. It is unclear how EMT is regulated by the TGF-β-miR-181-CBX7 axis if validated, and the interaction and regulation between TGF-β, miR-181, CBX7, and EMT is likewise unclear (Figure 2). Answering these questions will deepen our understanding of this axis and may provide new targets for intervention.

Once a significant miRNA and its critical targets are identified to play critical roles in the progression of HCC, several approaches can block miRNA activity, such as anti-miRs and blockmiRs [59,135,199,200,201]. Anti-miRs are antisense oligonucleotides with complementary bases that pair with the miRNA target, leading to miRNA silencing, mainly by sterically blocking the target miRNA [201]. One miRNA can target many, even hundreds, of mRNAs. Thus, it is very challenging for the anti-miR to prevent one specific mRNA only from miR-induced inhibition, as it will target many other mRNAs. BlockmiRs use antisense technology to reduce miRNA activity by specifically binding to the target mRNA rather than miRNA [59,201]. Whether it can be used for miR-181 and its specific targets is yet to be determined.

## Figures and Tables

**Table 1:** The miR-181 family in HCC ^1,2^.

Member	Expression	Target	Effect	Reference
miR-181a	Up	CBX7	pro-tumoral	[14,60] *
miR-181a	Up	PTEN	pro-tumoral	[155]
miR-181a	Up	E2F5	pro-tumoral	[154]
miR-181a-5p	Up	mt-CYB, mt-CO_2_	pro-tumoral	[156]
miR-181b/d	Up	TIMP3	pro-tumoral	[19]
miR-181b-5p	Up	TIMP3	pro-tumoral	[75]
miR-181a/b	Up	TIMP3, RASSF1A, NLK	pro-tumoral	[17]
miR-181a/b	Up	unknown	pro-tumoral	[157]
miR-181a/b/c	Up	CDX2, GATA6, NLK	pro-tumoral	[16,118,158]
miR-181a/b/d	Up	MKP-5	pro-tumoral	[153]
miR-181c-5p	Up	Fbxl3, SPAG9	pro-tumoral	[159,160]
miR-181a-5p	Down	c-Met	anti-tumoral	[161]
miR-181a-5p	Down	IGF2, CCNE1	anti-tumoral	[162]
miR-181a-5p	Down	Egr1	anti-tumoral	[163]
miR-181a	Up	RASSF1	drug resistance	[164]
miR-181a/d	Up	unknown	drug resistance	[165]
miR-181a-5p	Up	unknown	drug response	[18]
miR-181a		Sirt1	hepatic insulin sensitivity	[141]
miR-181b		Sirt1	hepatic steatosis	[139]
miR-181b-5p	Up	PTEN	activating HSC	[96]
miR-181b-5p	Up	TIMP3	activating HSC	[166]

^1^ This table does not list all studies. ^2^ It also includes a few of non-HCC papers, which may be helpful for understanding roles of miR-181s in HCC. * Tissue-specific miR-181ab1 knockout mice were used.
